# A Comparative Metabolomics Study of the Potential Marker Compounds in Feces from Different Hybrid Offspring of Huainan Pigs

**DOI:** 10.3390/ani14223282

**Published:** 2024-11-14

**Authors:** Yufu Li, Mingyang Jia, Junfeng Chen, Fujiu Liu, Qiaoling Ren, Xiangzhou Yan, Baosong Xing, Chuanying Pan, Jing Wang

**Affiliations:** 1 Henan Key Laboratory of Farm Animal Breeding and Nutritional Regulation, Henan Pig Breeding Engineering Research Centre, Institute of Animal Husbandry, Henan Academy of Agricultural Sciences, Number 116, Hua Yuan Road, Jinshui District, Zhengzhou 450002, China; liyufu2022@126.com (Y.L.); jia1my@163.com (M.J.); afeng008@163.com (J.C.); 13838262690@163.com (F.L.); renql@163.com (Q.R.); yanxiangzhou@163.com (X.Y.);; 2 College of Animal Science and Technology, Northwest A&F University, Ministry of Agriculture, Number 22, Xi Nong Road, Yangling 712100, China; panyu1980@126.com

**Keywords:** Huainan pig, fecal metabolite, untargeted metabolomics, LC-MS, hybridization

## Abstract

As a notable native Chinese genetic population, the Huainan pig has an exceptional meat quality but a low percentage of lean meat and subpar genetic performance. To better exploit the superior genetic traits of the Huainan pig and address knowledge gaps regarding the optimization of its hybrid offspring, this study used Huainan pigs as the maternal line and bred them with Yorkshire, Landrace, and Berkshire sires. This approach produced three hybrid combinations: Yorkshire × Huainan (YH), Landrace × Huainan (LH), and Berkshire × Huainan (BH). The body size, fat ratio, and average backfat thickness of these hybrid progeny were evaluated under the same feeding management and nutritional circumstances. The results revealed that the average backfat thickness of YH was significantly lower than that of LH and BH. In order to better understand the causes of these variations, fecal samples were taken from three pigs in each group for metabolomic analysis. A total of 2291 metabolites were identified, including benzene derivatives (16.6%), amino acids and their metabolites (14.5%), and organic acids (13.4%), with pyruvaldehyde and norethindrone acetate elevated in YH compared to LH and BH. In addition, the three hybrid pig groups commonly exhibited differences in the “glycerophospholipid metabolism” pathway. This variation may also contribute to differences in their fat ratio and backfat thickness. Our findings provide a novel perspective on the role of hybrid vigor in advancing the genetic population of Huainan pigs, while also revealing the unique metabolic characteristics of the YH with regard to fat deposition. This study is expected to enhance the conservation and effective utilization of genetic resources within the Huainan pig population.

## Simple Summary

Globally, pork is the meat consumed most on a per capita basis, and with the continuous growth in demand for meat, the quality of meat is being considered as an important indicator of the quality of pork products. However, the quality of pork often varies among different breeds of pigs. Huainan pigs, for example, have delicious meat but suffer from a low percentage of lean meat and poor reproductive performance. In order to improve the genetic traits of the local Huainan pig breed, our research utilized hybrid breeding techniques to cultivate new genetic populations with excellent meat quality. We crossed Huainan sows with pigs of three different foreign breeds and collected feces from the offspring in order to perform metabolomic sequencing, detect differences between genetic populations, and identify marker metabolites for the hybrid pigs. This study is expected to enhance the quality of Huainan pork and pork products.

## 1. Introduction

The quality of pork meat can be regulated by complex quantitative factors such as genetic, environmental, and nutritional factors [1]. The traits of different pig breeds vary significantly [2]. Western commercial pig breeds from Europe and America are renowned for their exceptional performance, being characterized by rapid development, outstanding feed conversion efficiency, and high carcass output [3]. Indigenous Chinese pig breeds typically have a superior meat quality in comparison to commercial Western pig breeds [4]. Nevertheless, the development of Chinese native pig breeds is sluggish, the rate of lean meat production is insufficient, and the ratio of feed to meat is poor, leading to limited economic advantages and market competitiveness [5]. Consequently, the primary approach to developing new populations in China involves the use of heterosis and the crossbreeding of Western breeds with native Chinese pigs [6].

Furthermore, gut microbes play a vital role in preserving the quality of pork by producing functional metabolites such as bile acids and short-chain fatty acids (SCFAs). These metabolites regulate the expression of genes and proteins related to fat synthesis and catabolism, thereby influencing intramuscular fat deposition and ultimately affecting the quality of pork [7]. Feces are commonly used as biological samples to capture gut microbes and metabolites [8]. Previous studies have demonstrated that the composition of gut metabolites is largely reflected by the fecal metabolome [9]. The analysis and characterization of these metabolites may provide information that is useful for the control and assessment of the quality of meat [10]. The techniques most often used for the identification and analysis of various fecal metabolites include gas chromatography, liquid chromatography, and tandem mass spectrometry [11,12,13]. Among these methods, the combination of liquid chromatography and high-resolution mass spectrometry (LC-MS) is extensively used for untargeted metabolomics due to its exceptional sensitivity, specificity, and wide detection capabilities [14].

Multiple studies have provided evidence that hybrids may have a beneficial impact on the microbiota and metabolism of pigs [15]. Previous studies have shown that, between native Chinese Jiaxing black (JXB) pigs and Duroc × Duroc × Berkshire × JXB (DDBJ) pigs, DDBJ pigs have remarkably greater levels of polyunsaturated fatty acids than JXB pigs [16]. In a different study, the metabolome of meat from five different crossbred pigs was evaluated. The researchers discovered that crossbreeding had a significant impact on amino acids such as alanine, carnosine, isoleucine, methionine, phenylalanine, and valine, as well as lactate, inosine monophosphate (IMP), inosine, glycerol, and compounds that contain choline [17]. Overall, these findings provide useful information and groundwork for enhancing the meat quality of native Chinese pigs, which may be achieved via hybrid breeding.

The Huainan pig is a renowned native breed in China that is famous for its significant accumulation of intramuscular fat; however, its development is slow and it has a low percentage of lean meat [18]. Therefore, researchers have consistently carried out crossbreeding operations to enhance the conservation and economic use of the Huainan pig [19]. However, no research has been conducted to investigate the variations in body performance and fecal metabolites of the offspring that result from the hybridization of Large Yorkshire, Landrace, and Berkshire pigs with Huainan swine. To fill the above research gaps, we conducted a comprehensive evaluation of the body traits of these three hybridized groups. Additionally, we performed a LC-MS/MS-based metabolomics study to evaluate the differences in the fecal metabolites of the three hybrid groups.

## 2. Materials and Methods

### 2.1. Ethics Statement

The experimental methods were performed in accordance with the Good Experimental Practices guidelines that are adopted by the Institute of Animal Science [20]. Additionally, all experimental protocols were approved by the Institute of Animal Science of the Henan Academy of Agricultural Sciences (code 2 May 2015) [21].

### 2.2. Hybrid Experiment Design

**Figure 1:**
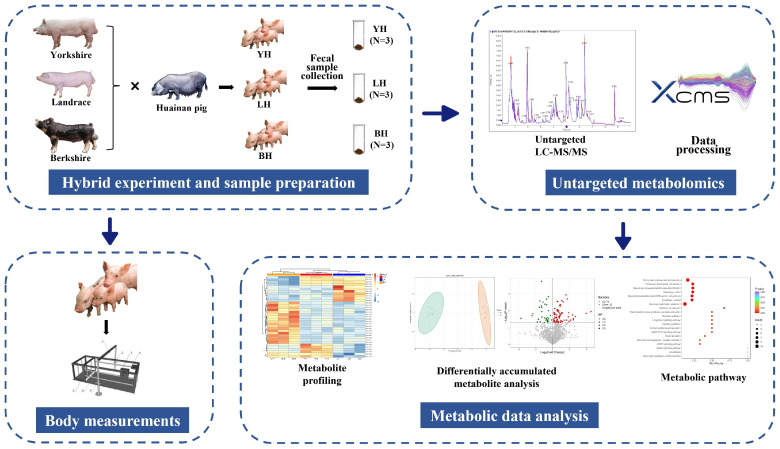
The overall workflow of the hybrid experiment and metabolomics strategy.

The hybrid experiment took place in the foundation seed farm of Huainan pigs owned by Henan Xingrui Agricultural and Animal Husbandry Technology Co., Ltd. (Xinyang, China). The experiment utilized a single-factor test, where 60 Huainan sows with a similar daily age, body condition, and fetal times were carefully chosen. The participating sows were divided into three groups of 20 based on their litter size, daily age, body weight, and randomized grouping. Each group was then bred with semen from Yorkshire, Landrace, and Berkshire boars, respectively (Figure 1). All the pigs were reared under the same conditions, with unrestricted access to both feed and water. The experimental feed formulation comprised 58% corn, 14% soybean meal, 9% wheat bran, 15% grass meal, and 4% premix. The diet provided a digestible energy content of 11.33 MJ/kg and contained 13.13% crude protein (Table 1). All other nutritional components conformed to the specified feeding standards (NY/T 65-2004) [22]. None of the sows and piglets were administered antibiotics or any other drugs throughout the experiment.

The premix provided the following compounds per kg diet: VA 10,800 IU; VB_1_ 10 mg; VD_3_ 3000 IU; VE 80 mg; VK_3_ 3000 IU; VB_12_ 20 mg; biotin 200 mg; *D*-pantothenic acid 15 mg; nicotinic acid 10 mg; Fe (as ferrous sulfate) 90 mg; Cu (as copper sulfate) 25 mg; Zn (as oxide zinc) 100 mg; and Mn (as manganese sulfate) 15 mg.

### 2.3. Animals and Sample Preparation

In this experiment, 30 castrated boars weighing around 30 kg each were chosen; these boars had similar body weights, daily ages, and body conditions. These boars were progeny from three hybrid combinations: Yorkshire × Huainan (YH), Landrace × Huainan (LH), and Berkshire × Huainan (BH) (Figure 1). When the pigs reached a market-standard body weight of approximately 100 kg, detailed developmental trait measurements were performed. These measurements included an accurate recording of the boars’ body weight, body height, and body length in order to assess their overall body size. The boars’ chest girth was measured to evaluate their thoracic development. Additionally, to facilitate an in-depth metabolomic analysis, three pigs were randomly selected from each group and fresh fecal samples were collected from the rectum using sterile swabs [23]. This procedure was carried out carefully to ensure that the samples were sterile and representative. Once these measurements had been performed, the pigs were slaughtered. The fat percentage was then measured, serving as a direct indicator of the meat’s composition and nutritional value [24]. Additionally, tissue samples were collected from the longissimus dorsi muscle on the left side of the carcass to assess the average backfat thickness [25].

### 2.4. Untargeted Metabolomics Profiling

Untargeted metabolomics services were provided by Frasergen Co., Ltd. (Wuhan, China). The samples, which were stored at −80 °C in a freezer, were thawed on ice. Once a 20 mg sample had been obtained, homogenization and extraction were performed. After centrifugation, 200 μL aliquots of the supernatant were transferred for LC-MS analysis. All samples were analyzed using two LC-MS methods (Figure 1). One aliquot was analyzed using positive ion conditions and was eluted with a T3 column (Waters ACQUITY Premier HSS T3 Column 1.8 µm, 2.1 mm × 100 mm; Waters, UK), using 0.1% formic acid in water as solvent A and 0.1% formic acid in acetonitrile as solvent B [26]. The analytical conditions were as follows: the column temperature was 40 °C; the flow rate was 0.4 mL/min; and the injection volume was 4 μL [27]. Another aliquot was analyzed under negative ion conditions by using the same elution gradient as the positive mode. Data acquisition was performed using the information-dependent acquisition (IDA) mode and Analyst TF 1.7.1 Software (Sciex, Concord, ON, Canada) [28].

### 2.5. Statistical Analysis

The original data file acquired via LC-MS was converted into mzXML format using ProteoWizard v.3.0.22 software (Palo Alto, CA, USA). The metabolite data were log2-transformed for statistical analysis in order to improve normality and were normalized. Metabolites from 9 samples were used for hierarchical cluster analysis (HCA) and orthogonal partial least squares discriminant analysis (OPLS-DA) using R v.4.3.3 software (ComplexHeatmap v.2.9.4, Heidelberg, Germany; MetaboAnalystR v.1.0.1, Montreal, Canada) to study metabolite accession-specific accumulation [29]. The *p* and fold change values were set to 0.05 and 2.0, respectively. Volcano graphs and upset plots were used to illustrate the number of differential metabolites. The metabolites identified were annotated using the KEGG Compound database “http://www.kegg.jp/kegg/compound/ (accessed on 17 August 2024)”, and the annotated metabolites were then mapped to the KEGG Pathway database “http://www.kegg.jp/kegg/pathway.html (accessed on 17 August 2024)” [30]. Significantly enriched pathways were identified using a hypergeometric test’s *p*-value for a given list of metabolites. All data were graphed using GraphPad Prism v.6.01 (GraphPad Software Inc., La Jolla, CA, USA) and R software [31].

## 3. Results

### 3.1. Comparison of Body Measurements of Three Hybrid Pig Genetic Populations

**Figure 2:**
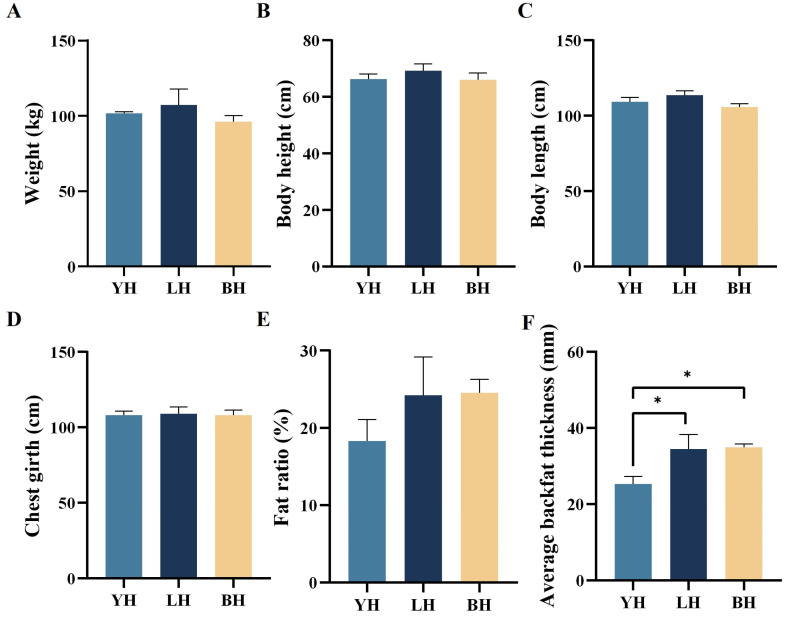
Comparative analysis of the three hybrid pig combinations in terms of weight (**A**), body height (**B**), body length (**C**) chest girth (**D**) fat ratio (**E**), and average backfat thickness (**F**). “*” means: *p* < 0.05.

All three hybrid pig genetic populations were reared simultaneously under uniform feeding conditions, including unrestricted access to food and water, until they reached the optimal market weight. Our study thoroughly recorded the growth characteristics of these pigs and found that the LH breed exhibited a greater body weight, height, length, and chest girth compared to the other hybrid breeds. However, these differences were not statistically significant, as illustrated in Figure 2.

In terms of the pigs’ fat ratio, a distinct distribution pattern was observed among the breeds: BH had the highest percentage of fat, followed by LH. Meanwhile, YH had a relatively lower percentage of back fat. A further analysis of the average backfat thickness revealed a similar trend, with BH having the highest and YH the lowest values. The average backfat thickness of YH was significantly lower than that of both BH and LH (*p* < 0.05).

### 3.2. Major Metabolites Profiling

**Figure 3:**
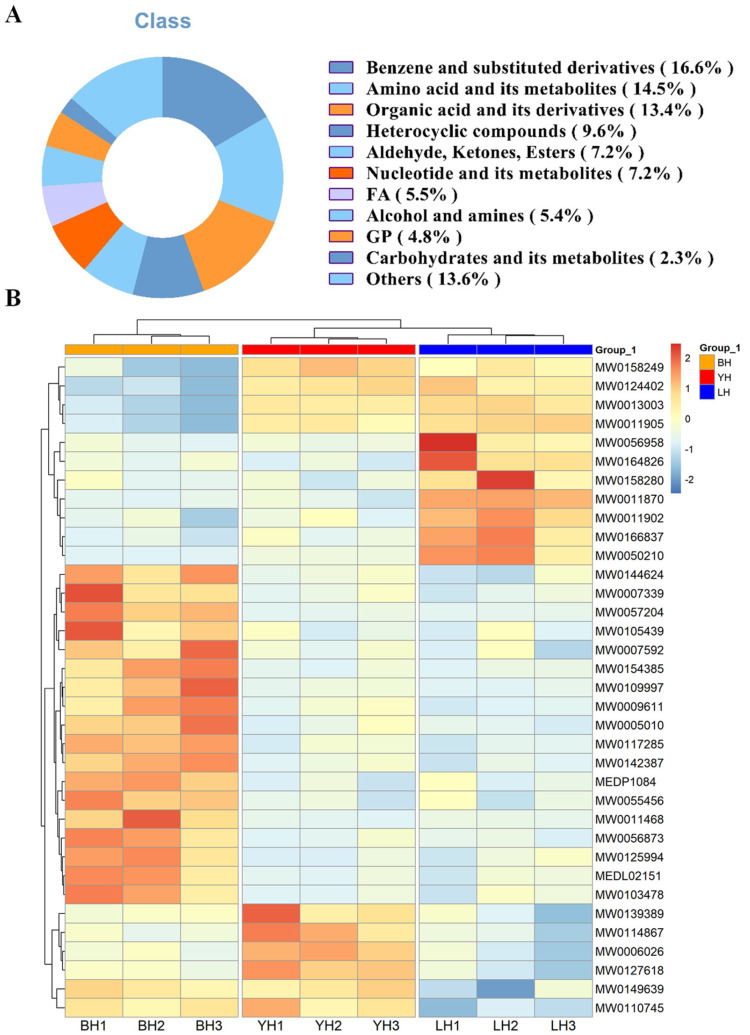
Composition (**A**) and clustering analysis heatmap (**B**) of metabolites in feces from three hybrid pig combinations. Colors correspond to the distinct values achieved following relative content normalization (red denotes high levels and green denotes low levels).

In order to understand the metabolic alterations that occur in the feces of various hybrid genetic populations, a thorough examination of the metabolites in these three hybrid pig genetic populations was carried out by utilizing untargeted metabolomics with LC-MS. A comprehensive analysis revealed a total of 2291 metabolites, including 766 metabolites in the negative ion mode and 1525 metabolites in the positive ion mode (Table S1). Specifically, these metabolites included 368 benzene and substituted derivatives (16.6%), 321 amino acids and their metabolites (14.5%), 297 organic acids and their derivatives (13.4%), 212 heterocyclic compounds (9.6%), and various additional chemicals (Figure 3A). A quantitative cluster analysis identified distinct metabolomic differences among YH, LH, and BH, with BH and LH showing lower concentrations of pyruvaldehyde and norethindrone acetate compared to YH (Figure 3B).

### 3.3. Differentially Accumulated Metabolite Analysis

**Figure 4:**
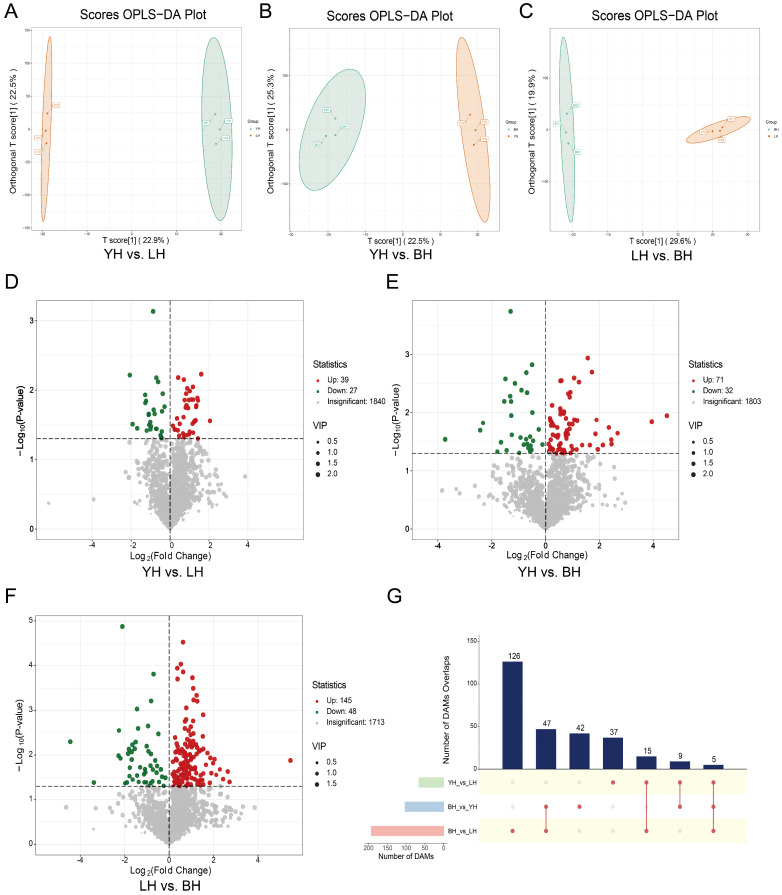
Investigation of DAMs across the three distinct hybrid pig combinations. Fecal metabolite profiling was performed using OPLS-DA models between (**A**) YH and LH, (**B**) YH and BH, and (**C**) LH and BH participants. Volcano graphs (**D**–**F**) showing the DAMs for the three groups. (**G**) Upset plots showing the overlapping and accession-specific DAMs.

Fecal metabolite samples from three hybrid pig combinations were compared pairwise to determine the differentially accumulated metabolites (DAMs). In the OPLS-DA models (Figure 4A–C), YH and LH clearly separated from BH, and LH separated from YH. This suggests that there are significant differences in the properties of the fecal metabolites of various hybrid pig combinations.

All 2291 metabolites were subsequently evaluated for DAMs using fold change (FC ≥ 2 or ≤0.5), the statistical significance of inter-group differences (*p* < 0.05), and the variable importance in the projection (VIP > 1) scores. The screening findings are shown graphically using volcano plots (Figure 4D–F) and upset plots (Figure 4G). For YH, there were 66 DAMs (39 upregulated and 27 downregulated) compared to LH. For BH, there were 103 DAMs (71 upregulated and 32 downregulated) compared to YH. For LH, there were 193 DAMs (145 upregulated and 48 downregulated) in comparison to BH (Table S2). These differential metabolites primarily belonged to amino acids and their metabolites, organic acids and their derivatives, glycerophospholipids (GPs), fatty acids (FAs) and glycerolipids (GLs) (Table S3).

Consistent with the clustering analysis, the levels of pyruvaldehyde and norethindrone acetate were significantly upregulated in the YH breed compared to BH and LH. Furthermore, metabolites such as succinic anhydride, 2,2-diphenylglycine, and (-)-callocatechin were notably upregulated in YH. Figure 4G shows that there was a discernible difference in the accumulation of five metabolites across the three groups. The differential metabolites commonly identified across all three groups included fumitremorgin, UDP-xylose, spiramycin, and others.

### 3.4. Differences in the Metabolic Pathway Between YH, LH, and BH Pigs

**Figure 5:**
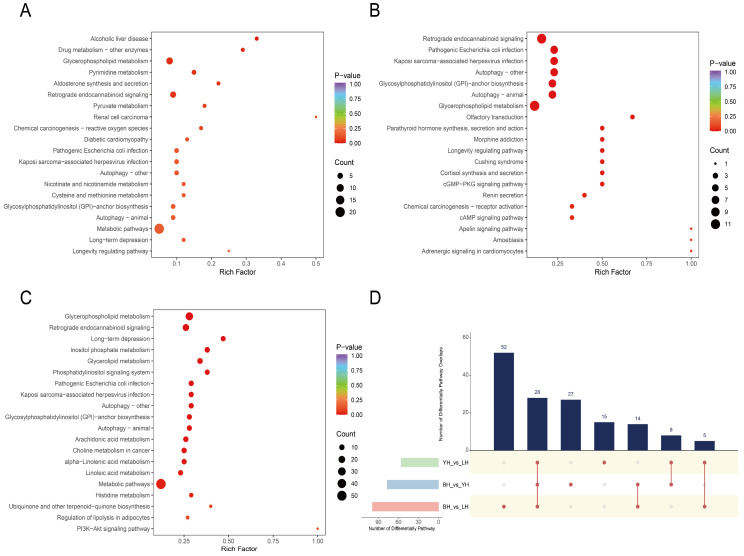
The KEGG enrichment plots show the metabolic pathways enriched with specific metabolites that are expressed differently between YH and LH (**A**), YH and BH (**B**), and LH and BH (**C**). The *x*-axis signifies the Rich Factor associated with each pathway, while the *y*-axis shows the names of the pathways arranged in order of their *p*-value. The color of the data points reflects the size of the *p*-value, where red shades suggest a higher level of enrichment. The magnitude of the data points corresponds to the quantity of metabolites that are differentially expressed and enriched in that particular pathway. (**D**) Upset plots showing the overlapping pathways.

The functions of these DAMs were determined by the KEGG pathway analysis (Figure 5A–C). In contrast with YH, the distinct metabolites in LH and BH mostly pertain to “glycerophospholipid metabolism” and “retrograde endocannabinoid signaling”. The metabolites that differ between LH and BH are mainly involved in “glycerolipid metabolism”, “inositol phosphate metabolism”, and “glycerophospholipid metabolism”. An overlapping enrichment of twenty-eight metabolic pathways was observed across the three groups (Figure 5D). The enrichment of the “glycerophospholipid metabolism” in all three groups suggests that this specific metabolic pathway plays a vital role in the physiological processes of these three hybrid pig genetic populations. This may be attributed to variations in metabolism among breeds and possibly corresponds to certain common biological functions or physiological processes.

## 4. Discussion

Globally, pork is the meat consumed most on a per capita basis [32]. Pork meat offers a significant amount of protein, provides several essential micronutrients, is reasonably priced, and is customarily accepted in most cultures [33,34]. Due to a continuous increase in the global demand for meat, the quality of pork meat has been recognized as a significant determinant of customer preferences. The quality of pork meat, as a multifaceted property, is affected by several physicochemical properties, including its pH value, tenderness, color, intramuscular fat concentration, fatty acid composition, and sensory qualities [35]. Additionally, the quality of pork produced by various pig strains is often inconsistent. Consequently, hybrid breeding techniques are frequently implemented in the field of animal husbandry to cultivate novel breeds with superior meat quality [36]. 

The Huainan pig is a highly commendable indigenous breed in China that is mostly found in the higher regions of the Huaihe River. It is known for its exceptional heat tolerance, resilience to harsh feeding, and substantial accumulation of fat inside the muscles [18]. It was officially included in the list of “Henan Local Excellent Livestock and Poultry Breeds” in 1986 and is categorized as the Huaihe River Black Pig in the “Chinese Pig Breed Encyclopedia” [37]. Nevertheless, Huainan pigs have a low percentage of lean meat. Therefore, researchers are aiming to improve the percentage of lean meat in Huainan pigs via hybridization. For example, one study used Yorkshire, Landrace, and Duroc pigs as sires and Huainan pigs as dams for crossbreeding, and the research results indicated that the hybridization process enhanced the average daily weight gain, lean meat ratio, and feed-to-meat ratio of the progeny generation [38]. 

To gain deeper insights into the differences among offspring from various Huainan pig crossbreeding combinations, we selected three representative crossbred pig types and focused on comparing their growth performance and fecal metabolic profiles. In our study, we chose to mate Huainan pigs with Yorkshire, Landrace, and Berkshire breeds due to their genetic characteristics and hybrid vigor. Yorkshire pigs are well known for their rapid growth and high rate of feed conversion, whereas Landrace pigs are recognized for their superior maternal traits and production of lean meat [39]. In contrast, Berkshire pigs are prized for their exceptional meat quality and flavor [40]. By crossbreeding these breeds with Huainan pigs, we aimed to harness their advantageous genetic traits to enhance the percentage of lean meat, growth rate, and overall meat quality of the offspring, while retaining the distinctive characteristics of the Huainan breed. A comparison of the body measurements of the three hybrid pig breeds revealed that YH pigs had a significantly lower average backfat thickness than BH and LH pigs. Additionally, YH exhibited the lowest fat ratio among the groups, indicating a potentially higher lean meat yield. However, YH exhibited slower growth and a smaller body size compared to BH and LH. Since all groups were raised under identical feeding conditions, we suppose that genetic differences may underlie these variations in nutrient intake and absorption; further investigations should therefore be performed to validate this assumption.

A further analysis of the fecal metabolism of the three pig groups revealed several significant differences between the YH, LH, and BH pigs (Figure 3, Figure 4 and Figure 5). Pairwise comparisons were conducted on fecal metabolite samples from the three hybrid pig combinations to identify differentially accumulated metabolites. Notably, five metabolites exhibited significant differences across all three groups, including fumitremorgin, UDP-xylose, and spiramycin. Among them, the UDP-xylose levels in YH exhibited a marked reduction compared to those in BH and LH. UDP-xylose serves as a crucial intermediate not only in xylose biosynthesis but also as an essential sugar donor in the synthesis of glycoproteins, polysaccharides, various metabolites, and oligosaccharides across plants, vertebrates, and fungi. This observed decrease in UDP-xylose in YH may suggest underlying variations in the sugar metabolism pathway between YH and the other groups, BH and LH [41]. However, the relationships between these metabolites and traits related to pig growth and meat quality currently remain unexplored. 

Additionally, our analysis revealed that the concentration of pyruvaldehyde and norethindrone acetate were greater in YH compared to BH and LH. Pyruvaldehyde belongs to the class of organic compounds known as alpha ketoaldehydes, which play notable roles in cellular signal transduction and gene expression regulation. Furthermore, pyruvaldehyde has been closely linked to the onset and progression of multiple diseases, highlighting its potential importance in pathophysiological mechanisms [42]. An investigation using untargeted metabolomics to determine the marker molecules in refrigerated pork revealed that pyruvaldehyde is a significant marker component linked to the freshness of refrigerated pork [43]. Norethisterone acetate, as a progestin drug, plays an essential role in the dynamic balance of fat deposition. It exerts this effect by finely regulating the activity of key enzymes involved in fat metabolism, significantly influencing adipocyte differentiation and proliferation, and optimizing fat tissue distribution. Scientific studies have revealed that the combination of 17β-estradiol (E2) and norethindrone acetate can significantly reduce fat deposition in postmenopausal women [44]. Another study investigated the effects of conventional doses of norethindrone acetate on the concentration and rate at which plasma triglycerides are secreted in the splanchnic region of pigs. The study found that norethindrone acetate inhibited the secretion of liver triglycerides in pigs fed with glucose, thereby exhibiting a hypolipidemic effect [45]. In light of the significantly elevated levels of norethindrone acetate observed in YH pigs compared to BH and LH pigs, we propose that norethindrone acetate may play a pivotal role in regulating lipid metabolism. Through this regulatory function, it may effectively reduce fat accumulation or promote fat breakdown in YH pigs, thereby contributing to a lower overall fat ratio and decreased average backfat thickness.

Several GPs and GLs are among the metabolites that are expressed differently. These phospholipid molecules can be involved in controlling the quality of meat via internal metabolism, hydrolysis, and oxidation [46]. Furthermore, the “glycerophospholipid metabolism” pathway, identified as a common differential metabolic pathway among the three groups, was significantly enriched in the various pig breeds. This pathway might have important functions related to the digestive absorption, growth metabolism, and immune disease resistance of the hybrid pigs. Glycerophospholipid metabolism is the primary mode of intramuscular fat accumulation in livestock and poultry production. Differential intramuscular fat accumulation in pigs may be attributed to changes in fatty acids, glycerol lipids, and glycerophospholipid metabolic pathways, as indicated by various studies [47]. We hypothesize that the variations in the “glycerophospholipid metabolism” levels may have been caused by genetic variations deriving from breed differences or variations in the intestinal microbiota, as the three breeds of pigs were all subjected to the same management practices and nutrition levels. Prior research has shown a correlation between variations in the lipid and metabolic pathways in pork and different pig breeds, which aligns with the findings of this study [48,49].

In summary, we analyzed the different metabolites and metabolic pathways associated with the fecal samples of YH, LH, and BH in the hybrid Huainan pig line using untargeted metabolomics technologies. Our results revealed that hybridization reshaped the fecal metabolome. Nevertheless, the factors contributing to the variations in the fecal metabolome among each group were complex. The feeding conditions and dietary content of the pigs in each group were the same, and their ages were comparable. Hence, we assume that hybridization is the primary factor contributing to the variations in the fecal metabolome across the groups. This study assessed differences among offspring from various crossbreeding combinations, yet several areas warrant further exploration. Future research could extend beyond the current framework to investigate metabolomic differences between purebred Huainan pigs and their crossbred offspring. Expanding the study to include additional pig breeds may also uncover valuable biomarkers, enriching the scientific foundation for genetic improvement and breeding strategies. Thus, we plan to continue this investigation in our future work.

## 5. Conclusions

A comparative analysis of the growth and meat quality of the three hybrid Huainan pig breeds revealed that the average backfat thickness of YH pigs was significantly reduced compared to that of BH and LH pigs. Furthermore, LC-MS/MS metabolomics was successfully used to analyze the different metabolites and metabolic pathways of the fecal samples in the three hybrid pig breeds. As a result, a comprehensive metabolomic profile consisting of 2219 metabolites was identified. A pairwise analysis was performed on the fecal metabolite samples obtained from the three different hybrid pig breed combinations. The results showed notable differences in the fecal metabolite profiles across the various hybrid pig combinations. In addition, pyruvaldehyde, norethindrone acetate, and “glycerophospholipid metabolism” were identified as crucial marker compounds and metabolic pathways related to the differences between YH pigs and the BH and LH breeds. This research presents a thorough analysis of the fecal metabolites that could be used to find marker metabolites in hybrid pigs, with the potential to improve the quality of the meat and meat products provided by Huainan pigs.

## Figures and Tables

**Table 1:** Composition and nutrient levels of the basal diet (air-dried).

Ingredients	Contents	Nutrient Components	Contents
Corn	58.00	CP	13.13
Soybean meal	14.00	DE/(MJ/kg)	11.33
Wheat bran	9.00		
Grass meal	15.00		
Premix	4.00		
Total	100.00		

## Data Availability

The data presented in the study are deposited in the figshare database (https://doi.org/10.6084/m9.figshare.27093889.v2).

## Supplementary Materials

The following supporting information can be downloaded at: https://www.mdpi.com/article/10.3390/ani14223282/s1, Table S1: Information of metabolites detected in different ion modes; Table S2: Type of differentially accumulated metabolites; Table S3: Number of differentially accumulated metabolites class.
